# Induced spawning of siban fish *Cyclocheilichthys apogon* using Ovaprim

**DOI:** 10.12688/f1000research.12885.1

**Published:** 2017-10-18

**Authors:** Nuraini Nuraini, Afrizal Tanjung, Trisla Warningsih, Zainal Abidin Muchlisin

**Affiliations:** 1 Department of Aquaculture, Faculty of Fisheries and Marine Sciences, Universitas Riau, Riau, Indonesia; 2 Department of Marine Science, Faculty of Fisheries and Marine Sciences, Universitas Riau, Riau, Indonesia; 3 Department of Socio-Economic Fisheries, Faculty of Fisheries and Marine Sciences, Universitas Riau, Riau, Indonesia; 4 Department of Aquaculture, Faculty of Marine and Fisheries, Syiah Kuala University, Banda Aceh, Indonesia

**Keywords:** fish breeding, freshwater fish, pituitary, (sGnRH), domperidone, hatchery

## Abstract

**Background**: The objective of the present study was to examine the effect of Ovaprim dosage on the latency period, relative number of ovulated eggs, fertilization, hatching, and survival rates of the siban fish, *Cyclocheilichthys apogon*.

**Methods**: Three dosages of Ovaprim were tested in this study, namely 0.3 ml kg-1 of broodfish body weight, 0.5 ml kg-1 body weight, and 0.7 ml kg-1 body weight, plus control (without Ovaprim).

**Results**: The results showed that the best latency period, relative number of ovulated eggs, fertilization, hatching, and survival rates were obtained at a dosage of 0.7 ml kg ^-1^ body weight.

**Conclusions**: The best dosage of Ovaprim for siban fish from the dosages tested, was determined to be 0.7 ml kg-1 body weight.

## Introduction

Siban fish *Cyclocheilichthys apogon* is a commercial freshwater fish in Indonesia. This species is distributed in South East Asia regions such as Sumatra, Borneo, Malaysia, and Mekong, Thailand ^1^. Siban fish is one of the main targets of local fishermen in Riau Province, Indonesia, resulting in decreasing wild populations over the last decade (Personal communication with local fishermen of the Kampar River). Therefore, the cultivation of siban fish needs to develop in relation to meeting the market demand, without disturbing the wild population.

Nurhusniah ^2^ and Sari ^3^ have studied the reproductive biology of the siban fish, but no other aspect has been studied yet. Nonetheless, the culturing of the siban fish has been initiated in Riau Province, Indonesia; the larvae were collected from the wild at low quality and quantity, with high seasonal dependence ^4^, resulting in low production. Therefore, the breeding technology of siban fish is crucially needed to overcome these problems and to support the aquaculture business of the local people.

Induced spawning is one of the common methods to stimulate ovulation of the fish in the hatchery. With this method, hormones are playing an important role ^5^. Several natural and artificial hormones have been used to induce breeding of fish, while Ovaprim is one of the most popular and effective solutions to stimulate the maturation of male and female broodfish. Ovaprim contains combinations of salmon gonadotropin-releasing hormone (sGnRH) and domperidone. These hormones have been applied successfully to induce spawning of seurukan fish *Osteochilus vittatus* ^6^, selais *Ompok hypophthalmus* ^7, 8^, common carp *Cyprinus carpio* ^9^, mali-mali *Labiobarbus festivus* ^10^, and lelan fish *Osteochilus pleurotaenia* ^11^.

Ovaprim has several advantages, for example, it is cheap, has many practical uses, and it is easy to find in the local market in Indonesia. However, Ovaprim has never been used to induce spawning of siban fish. Hence, the objective of the present study was to determine an effective dosage of Ovaprim for siban female broodstock.

## Methods

### Study timeline and site

The experiments were carried out within the ethical guidelines in animal research developed by NC3Rs. The study was conducted between April and June 2016, at the Fish Breeding Laboratory, Faculty of Fisheries and Marine Sciences, University of Riau, Pekanbaru, Indonesia. A total of 12 male and 12 female broodfish of the siban fish *Cyclocheilichthys apogon* species were collected from the Kampar River, Riau Province. The broodfish ranged between 10 and 15 cm in total length, and 18.42 and 31.15g in total weight. The fish were acclimatized for 24 hours prior to inducing with Ovaprim.

### Hormone administration

The experiment was carried out in compliance with the ethical guidelines provided by the Research Institution of Riau University (SOP/02/PL/LPPM/2016). Three dosages of Ovaprim (Syndel, Canada) were tested in this study: for females, 0.3 ml kg ^-1^ body weight (BW), 0.5 ml kg ^-1^BW, and 0.7 ml kg ^-1^BW; for males, 0.15 ml kg ^-1^BW. The broodfish in control groups were injected with physiological solution (0.9% NaCL) at the dosage of 0.25 ml kg ^-1^BW.

### Sperm and egg collection

The female broodfish were injected with their respective dosage two times; the first injection was at 8.00 PM with half of the tested dosage, and the second injection was conducted 6 hours after the first injection (at 2.00 AM), with the remainder of the tested dosage. The female broodfish was ovulated 6 hours after the second injection (at 8.00 AM). The eggs were collected by gentle finger pressure to the abdomen. The collected eggs were put in a plastic jar and kept in an ice box at 4°C ^6^.

The males were injected with a single dose of 0.25 ml kg ^-1 ^BW at 8.00 PM. The male siban fish was sacrificed with MS-222 (Merck), prepared by dissolving 4g of MS-222 in 5L tap water ^12^. The testes were removed and washed with physiological solution and then perforated and chopped with scissors. The semen was gently squeezed out and put in a tube, and kept in an icebox (4°C). The semen was mixed with physiological solution (0.9% NaCl) at dilution ratio 1: 100 (v/v).

### Fertilization and incubation

A total of 1 ml eggs and 1 ml of diluted sperm were mixed homogenously in a plastic basin (Calista, Volume 500 mL) and with approximately 1 ml of fertilization solution (contains 4 g urea and 3 g NaCl L ^-1^) developed by The Laboratory of Fish Breeding, Faculty of Fisheries and Marine Sciences, Universitas Riau. The resulting mixture was left in contact for 5 minutes. The incubation basin was installed with 24-hour portable aerator and LED lamp.

A total of 100 eggs were taken randomly and then incubated in a plastic basin (Calista, volume: 2L), with three replicates at a water temperature of 25–27°C in a water heater. Successful fertilization was observed 8 hours after incubation. Unfertilized eggs were identified by their opacity; the unfertilized eggs were removed from the jar, while hatching rate was monitored at two-hour intervals ^13^. The larvae were fed on *Tubifex* sp. *ad libitum* on day 5 after being hatched and reared in the same jar for 40 days.

### Measured parameters and data analysis

The latency period, the relative number of ovulated eggs, egg diameter, fertilization rate, hatching rate, and survival rate of larvae on day 40 after hatching were measured. The latency period was determined by calculating the time between the second injection and ovulation. The relative number of ovulated eggs was calculated by dividing the total number of released eggs after Ovaprim injection by the total body weight of the female broodfish. Fertilization, hatching, and survival rates were calculated based on Muchlisin *et al* ^6, 14^ and Adami *et al* ^15^. All data were subjected to One-Way Analysis of Variance (ANOVA), followed by the Duncan multiple-range test.

## Results

The ANOVA test revealed that different Ovaprim dosages had a significant effect on latency period, relative number of ovulated eggs, fertilization, hatching, and survival rates (p<0.05). The result showed that the latency period was faster at a dosage of 7 ml kg ^-1^BW; this value was significantly different from other dosages. A higher relative number of ovulated eggs and fertilization, hatching, and survival rates were also recorded at the Ovaprim dosage 7 ml kg ^-1^ BW; these values are significantly different from other dosages ( Table 1). In general, the relative number of ovulated eggs, fertilization, hatching, and survival rates were increased with increasing Ovaprim dosage. However, there were no significant differences between the values seen at 3 ml kg ^-1 ^BW and 5 ml kg ^-1 ^BW dosage.

**Table 1.** Latency period, fertilization, hatching, and survival rate of siban fish *Cyclocheilichthys apogon* based on Ovaprim dosages.

No	Parameter	Ovaprim dosage (ml kg ^-1^ body weight)			
		0	3	5	7
1	Latency period (hour)	10.01±0.01 ^d^	7.27±0.06 ^c^	7.18±0.04 ^b^	7.07±0.04 ^a^
2	The relative number of ovulated eggs (eggs g ^-1^ of BW)	5.5±0.71 ^a^	10.67±6.03 ^b^	11.00±0.00 ^b^	19.00±11.53 ^c^
3	Fertilization rate (%)	40.00±1.41 ^a^	60.00±8.19 ^b^	60.00±7.55 ^b^	76.67±9.07 ^c^
4	Hatching rate (%)	25.55±2.12 ^a^	62.94±2.05 ^b^	56.95±5.31 ^b^	76.00±2.92 ^c^
5	Survival rate on day 40 after hatched (%)	40.00±2.83 ^a^	61.95±1.80 ^b^	58.60±1.08 ^b^	79.99±9.13 ^c^

Raw data collected for the latency period, relative numbers of ovulated eggs, fertilization, hatching and survival rates of the siban fish Cyclocheilichthys apogon Click here for additional data file. Copyright: © 2017 Nuraini N et al. 2017 Data associated with the article are available under the terms of the Creative Commons Zero "No rights reserved" data waiver (CC0 1.0 Public domain dedication).

## Discussion

The results indicate a decreased latency period at increased Ovaprim dosage. On the contrary, the relative number of ovulated eggs, fertilization, hatching, and survival rates were increased at increased dosage. The best results for all parameters were recorded at the Ovaprim dosage of 7 ml kg ^-1^ body weight. Therefore, the higher dosage of Ovaprim (7 ml kg ^-1^ BW) was an effective dosage in inducing spawning of the siban fish compared to the lower dosage (3 ml kg ^-^1 and 5 ml kg ^-^1). This is probably because the combination of sGnRH and domperidon in Ovaprim solution at higher doses of 7 ml kg ^-1^ BW stimulated gonadotropin (GTH II or LH) secretion in the pituitary gland of the broodfish to a greater extent, and then induced gonad maturation and ovulation. The higher levels of gonadotropin will induce ovulation faster ^16^. According to Ithisom ^9^, the hormone works optimally at a certain dose; and changing the dose will reduce effectiveness. Therefore, determining the optimum dose is crucial.

The influence of sGnRH and domperidon at the higher dosage most likely inhibited secretion of dopamine and stimulated the pituitary gland to secrete GTH. The GTH in this case, GTH II, would stimulate the theca cells to secrete hormone 17-alpha hydroxyprogesterone, which would then be converted into maturation-inducing steroid by the enzyme 20-beta-dihydroxy steroids, stimulating follicles to burst as the ooctes hydrate ^17, 18^.

The results showed that the high fertilization rate was obtained at doses of 0.7 ml kg ^-1^ BW; this dosage probably gave the optimum effect of sGnRH and domperidone to increase the quantity and quality of eggs. This is indicated by the higher number of ovulated eggs as recorded at this dosage. According to Nuraini *et al.* ^19^ the optimum dosage of stimulating hormone results in better ovulation and improves egg quality for the selais fish, *Ompok hypophthalmus*. Moreover, Woynarovich and Horvath ^20^ stated that the fertilization rate is strongly influenced by the quality of eggs and spermatozoa. Prostaglandin plays an important role in inducing ovulation and significantly influences fertilization and hatching rates. This is because prostaglandin contains arachidonic acid derived from essential fatty acids that determine the egg quality ^21^. In addition, the quality of eggs is influenced by several factors including the hormone levels ^22^, nutrition ^23^, genetics ^24^ and the environment ^25^. Bromage *et al.* ^26^ stated that the quality of eggs is reflected in higher fertilization and hatching rates.

Besides being influenced by hormonal factors, the hatching rate is also influenced by the temperature of the incubation media, dissolved oxygen, pH, and light intensity ^20^. In general, the survival rate of siban larvae was categorized as being at a good level. According to Alikunhi *et al*. ^27^ there are three levels of survival rate for the larvae: a good level when the survival is higher than 50%, moderate when between 30 and 50%, and low when the survival is lower than 30%. However, the survival of the larvae in this study was lower compared to seurukan fish *Osteochilus vittatus* and river catfish *Mystus nigricep,* which were injected with Ovaprim 0.5 ml kg ^-1^ BW, resulting in a survival rate of between 80.66 and 90% ^6, 28, 29^. It is assumed that the siban fish requires higher dosages of Ovaprim to induce gonad maturation as recorded in this study.

## Conclusions

It is concluded that different doses of Ovaprim had a significant effect on the latency period, the relative number of ovulated eggs, fertilization, hatching, and survival rates of siban fish. The best Ovaprim dosage, from the ones tested, was 0.7 ml kg ^-1^ BW.

## Data availability

The data referenced by this article are under copyright with the following copyright statement: Copyright: © 2017 Nuraini N et al.

Data associated with the article are available under the terms of the Creative Commons Zero "No rights reserved" data waiver (CC0 1.0 Public domain dedication). 

Dataset 1: Raw data collected for the latency period, relative numbers of ovulated eggs, fertilization, hatching and survival rates of the siban fish *Cyclocheilichthys apogon.* DOI, 10.5256/f1000research.12885.d180825 ^30^.
