# Supplementary material for: Induced spawning of siban fish Cyclocheilichthys apogon using Ovaprim
Source: F1000Res. 2017 Oct 18;6:1855. [Version 1] doi: 10.12688/f1000research.12885.1 (PMC5686476; doi:10.12688/f1000research.12885.1)
Supplement: Peer review history

# Peer review: Referee response for version 1

**DOI:** 10.5256/f1000research.13967.r27098

**Published:** 2017-11-14

**Authors:** Agung Damar Syakti

**Affiliations:** 1 Center for Maritime Biosciences Studies, Institute for Sciences and Community Service, Faculty of Fisheries and Marine Science, Jenderal Soedirman University, Purwokerto, Indonesia

This manuscript studied the effect of Ovaprim dosage on the latency period, relative number of ovulated eggs, fertilization, hatching, and survival rates of the siban fish, *Cyclocheilichthys apogon*.  Scientifically, overall quality of the manuscript is interesting and good. This work provides insight into the best latency period, relative number of ovulated eggs, fertilization, hatching, and survival rates of the optimum dosage of Ovaprim. Thus, from my point of view, this paper can provide some important scientific data and values. I can recommend the acceptance and indexing of this manuscript in *F1000Research*. However, there is some specific comment and queries to be considered and it is necessary to pay close attention to proper sentence structure and grammar in some sentences of the manuscript.

I have read this submission. I believe that I have an appropriate level of expertise to confirm that it is of an acceptable scientific standard.

# Peer review: Referee response for version 1

**DOI:** 10.5256/f1000research.13967.r27096

**Published:** 2017-11-14

**Authors:** Suparno Pranoto

**Affiliations:** 1 Faculty of Fisheries and Marine Sciences, University of Bung Hatta, Padang, Indonesia

**Title:**

 The title is good, brief and informative, you need to add the fish and the author name, also the year.

**Abstract:**

 The conclusion still states the result of the research

**Introduction:**

 In the introduction, give the information which is relevant with the Induced spawning of siban fish *Cyclocheilichthys apogon* using Ovaprim.

 Suggestion: The introduction should have information of  previous research  about siban fish reproduction with specifically,the latest reference and the novelty about the research.

**Methods:**

 Please add the research design.  Method of research must  be added with the clear reference.

**Results:**

 Please  describe the table  1 and emphasize the findings.  Findings on the latency period, relative number of ovulated eggs, fertilization, hatching, and survival rates of the siban fish.

**Discussion:** 

 Discussion must be based on the emphasis on the findings, and relation of the findings with the basic concepts. Is there any contradiction and suitability with other peoples research which is related with using ovaprim to siban fish?

**Conclusion** **:**

 Please write the conclusion briefly and clearly to answer the  purpose  of the research. Conclusion is not the result of the research.

**References:**

 Most of the references are published before 2012.  References should be taken from the last 5 years.

  An annotated Word version of the of the article is available here:

https://f1000researchdata.s3.amazonaws.com/linked/184593.Surpano_Edt-Siban_Fish_%28Nuraini%29.docx

I have read this submission. I believe that I have an appropriate level of expertise to confirm that it is of an acceptable scientific standard, however I have significant reservations, as outlined above.

# Peer review: Referee response for version 1

**DOI:** 10.5256/f1000research.13967.r27095

**Published:** 2017-10-30

**Authors:** Javad Ghasemzadeh

**Affiliations:** 1 Faculty of Marine Sciences, Chabahar Maritime University, Chabahar, Iran

This paper is the outcome of a well designed and executed research plan. It has clearly reviewed the current literature and background of the research, and the objectives of their research, and clear explanation of materials and methods which can be used and replicated in similar research work on different fishes. The statistical analysis applied for this type of research is simple, logical and interpreted very well. The results are given based on the clear and measured outcomes of the methods applied in the experiment which is applicable and reproducible in similar research work on the induced and artificial propagation of fishes (especially on freshwater fishes). The conclusions are clearly based on the measured and practically approved results, with no particular limitations being addressed. This article is good enough to be indexed with its present condition, and I did not find any problem in the English language used,.

I have read this submission. I believe that I have an appropriate level of expertise to confirm that it is of an acceptable scientific standard.
